# Physical Activity and Physical Fitness in Adolescents after the COVID-19 Lockdown and One Year Afterward

**DOI:** 10.3390/ijerph192214660

**Published:** 2022-11-08

**Authors:** Alejandro Carriedo, Jose Antonio Cecchini, Luis Enrique Fernández-Álvarez, Carmen González

**Affiliations:** Department of Educational Science, University of Oviedo, 33003 Oviedo, Spain

**Keywords:** confinement, pandemic, physical activity, sport, health

## Abstract

The purpose of this study was to analyze the changes in physical activity and physical fitness between the beginning of the first academic year after a confinement (November 2020) and the beginning of the second academic year after a confinement (“new normality”: November 2021) in a cohort of adolescents. Moreover, the evolution of physical fitness after controlling for physical activity was examined. A total of 687 students (*M* = 15.35, *SD* = 1.677) from a high school located in a rural town in northern Spain gave information on their physical activity (PA) levels in two different periods. Linear mixed models were used to examine these changes. The results indicated that vigorous physical activity (VPA) and the metabolic equivalent of task (MET) significantly increased between the two periods. A growth tendency of several components of fitness (upper body power, strength endurance, cardiovascular fitness, flexibility, and eye–hand coordination) was also observed. Finally, the results indicated that belonging to a sports club and getting involved in more VPA better explained the development in cardiovascular and muscle fitness between the two time points. Thus, the results of this study highlighted the relevance of membership in a sports club and vigorous PA in order to mitigate the potential negative effect of social distancing measures on physical fitness.

## 1. Introduction

On 7 January 2020, the World Health Organization (WHO) identified a new coronavirus that constituted a global health emergency. Experts determined that person-to-person transmission happened during the incubation period of the virus [1]. Therefore, all governments in the world established different measures to interrupt the chain of transmission, such as isolating infected persons and tracing those with whom they had interacted [2]. Thus, restrictive measures such as social distancing, home confinement, case detection, isolation, contact tracing, and the quarantine of those exposed were considered efficient actions to control the spread of this disease. In Spain, the government declared a state of emergency, which was extended from 14 March to 21 June 2020. Freedom of movement was limited to 50 days. Children were isolated in their homes until the end of April 2020. Afterward, different types of social distancing measures were imposed. For instance, the practice of physical sports activities was regulated; during the first year of the pandemic, no sports competitions or training could be undertaken. However, the spread of the virus did not stop, and from 25 October 2020 to 9 May 2021 a new state of emergency was declared. Less restrictive social distancing measures were then stated and a “new normality” period began. In this new period, sports competitions and training were allowed.

There is a strong link between physical activity and health [3,4]. For instance, a few studies have observed relationships between physical activity and health [5] and between physical fitness and health [6]. Adolescence is a critical period of human development that leads to adulthood. Physical activity is essential for children and adolescents [7], and the World Health Organization recommends that children accumulate an average of 60 min/day of moderate to vigorous physical activity (MVPA) across a week [7]. However, physical activity levels tend to decrease with age [8]. Several studies have observed that adolescents worldwide do not reach the physical activity recommendations [9,10]. This is even more worrisome if we take into account that the social distancing measures implemented during the COVID-19 pandemic could have resulted in a decrease in their physical activity levels; therefore, it is plausible that physical fitness has been affected [11]. A few studies found a reduction in physical activity due to confinement [12,13,14]. This decrease seemed to be more intense among children who used to participate in organized team sports [15].

Physical fitness levels are important predictors of health outcomes such as cardiovascular and skeletal health or mental wellbeing [16]. Particularly, cardiorespiratory fitness refers to the capacity of the circulatory and respiratory systems to supply oxygen to skeletal muscle for the energy production needed during physical activity; it is an important marker of the health indicators of physical health in youths [17]. Likewise, there is a link between cardiorespiratory fitness and mental health in adolescents [18], which also highlights the importance of being physically active during childhood. Cardiorespiratory fitness can be assessed through fitness tests such as the 20 m shutter run test, which measures aerobic cardiovascular endurance. A recent study in Spain observed that 89 adolescents did not change their cardiorespiratory fitness measured indirectly through a 20 m shuttle run test before and after the COVID-19 confinement [19]. However, other studies detected a decrease in a few physical fitness tests including cardiovascular endurance and muscle strength [20]. These studies compared the physical fitness of participants before and after the social distancing measures, but they did not control for other confounding variables such as the volume of physical activity that the participants were involved in during that period. Thus, the understanding of the impact of social distancing measures on the physical fitness of adolescents could be enhanced by studies that consider the variance in these types of variables.

Based on the above, one purpose of this study was to analyze the changes in physical activity between the beginning of the first academic year after a confinement (November 2020) and the beginning of the second academic year after a confinement (the so-called “new normality” period, November 2021) in a cohort of adolescents. A second aim was to investigate whether physical fitness varied between these two time measurement points. Finally, the third goal was to examine the evolution of physical fitness after controlling for physical activity levels and belonging to a sports club during this period.

## 2. Materials and Methods

### 2.1. Participants

The sample consisted of 687 compulsory education and baccalaureate students (375 males and 312 females), aged between 11 and 19 years old (*M* = 15.35, *SD* = 1.77) from a high school located in a rural environment in the north of Spain. The inclusion criteria were age ≥ 11 years old and enrolled in the high school that was selected to conduct the study; participants also had to declare that they were in good health. Those students who reported a physical limitation were exempted from performing the tests involving the affected parts.

### 2.2. Design, Instruments, and Data Collection

This was a quantitative within-subject study design. All participants took part in two measurement points (T1 and T2) and the outcomes were compared between them. A multilevel approach was used. In this study, physical activity levels and physical fitness were measured twice in a cohort of adolescents from a rural population (at the beginning of the first academic year after a confinement (November 2020) and at the beginning of the second academic year after a confinement (“new normality”: November 2021). First, approval was obtained from the ethics committee of the university where the study was conducted. Subsequently, the research was introduced to the administration team of the participating school and the parents of the students; written consent was obtained from all of them. Students filled out a questionnaire that included questions about physical activity and socio-demographic information. Several fitness tests were then used to measure six components of their physical fitness (aerobic cardiovascular endurance, upper and lower body power, strength endurance, speed, and flexibility) as well as their coordination. All tests used in this study belonged to the Eurofit fitness test battery. The physical fitness measurements were registered by the two physical education teachers of the high school, who had more than 10 years of experience in registering physical fitness measures. All tests were performed in the gym of the high school under the supervision of the teachers of the students at two time points (November 2020 and November 2021). In order not to influence the measurement process, each student was evaluated by the same evaluator at the two time points. Thus, each physical education teacher registered the physical fitness measures of their own students. All students were informed that the information would be treated confidentially. They were also informed that their participation was voluntary. The first wave of data was collected in the third week of November 2020, a period when physical activity and sports were regulated by the authorities and several activities were not allowed (e.g., sport competitions). The quarantine finished on 24 April 2020; from then, several stages of de-escalation measures were established such as a relaxed confinement or limitations to the practices and official tournaments of sports clubs. One year afterward, participants were asked again about the physical activity they were involved in and physical fitness was measured again (November 2021). The study respected the key ethical values and followed the principles of the World Medical Declaration of Helsinki.

### 2.3. Instruments and Variables

***Physical Activity***. The International PA Questionnaire (IPAQ) [21] is an instrument developed for the cross-national monitoring of PA and inactivity. In the present study, the Spanish-validated version (obtained from www.ipaq.ki.se accessed on 5 November 2022) of the short form (7-day recall) was used [22]. This is an appropriate outcome measure for clinical and research use [22]. This version provides information about the time spent involved in three physical activity (PA) intensity levels: (a) light (LPA); (b) moderate (MPA); and (c) vigorous (VPA). In addition, this instrument allows the calculation of the metabolic equivalent of task (MET). The MET is a system used to measure the energy requirements for PA. One MET corresponds with the equivalent of the energy needed for a basal metabolic rate. It has been stablished that LPA requires fewer than 3 METs, MPA requires between 3 and 5.9 METs, and VPA requires more than 6 METs.

***Other measures.*** The other measures included in the questionnaire were gender, age, and membership of a sports club (Yes, No).

***Trunk flexion***. Sit-and-reach (SAR) measured the flexibility of the trunk. Initial position: the barefoot performer was required to sit in front of a box with their legs fully extended and the soles of their feet in full contact with the wall of a box. Procedure: the subject bent their trunk forward with straight legs and extended their arms and the palms of their hands to a ruler, trying to reach as far as possible. Final position: the subject reached out and held that position for at least one to two seconds whilst the distance was recorded in centimeters and millimeters. The best of two results was registered.

***Standing long jump test (broad jump)***. This was a test of explosive leg power. Initial position: the performer was required to stand straight, with their feet slightly apart and their toes behind a starting line. Development: the participants had to gain momentum to jump by bending their legs and pushing with their arms from behind to in front; the participant then attempted to jump as far as possible, landing on both feet without falling backward. Final position: At the moment of landing, the performer had to reach a maximum distance without losing balance. Assessment of the test: The total jump distance was the sum of three component distances; the takeoff distance, the flight distance, and the landing distance. The best of two results was registered.

***Crunches in 30 s (A30S)***. Sit-ups (SUP) measured the strength endurance of the abdominal muscles. Initial Position: the performer was in a supine position with their legs bent at 90° and their feet slightly apart with their fingers intertwined behind their neck; an assistant held the feet to fix them to the ground. Procedure: upon hearing the start signal, the performer had to try to attempt as many repetitions as possible—always touching the knees with the elbows and their back on the mat—and the evaluator counted the number of repetitions aloud. Completion: when 30 s had passed, the observer indicated to the performer that the test had finished. Assessment of the test: the number of repetitions performed correctly was registered.

***Course Navette (CN)***. This 20 m shuttle run test (20 m SR) measured the aerobic cardiovascular endurance or cardiorespiratory fitness. Initial position: the participants were behind a starting line, one meter away from each other. Procedure: A tape recorder was started. Upon hearing a sound signal, the participants moved to the opposite line (50 m), crossing it and waiting for the next sound signal. The participants had to follow the rhythm marked by the tape recorder for as long as possible. The speed increased incrementally. The test ended when the participants could not reach the opposite line before the sound of the subsequent warning on two consecutive occasions. Evaluation of the test: the completed stages were registered and the last stage announced before the performers abandoned the test was registered.

***Medicine ball throw (3 kg)*** (MBT3K). The medicine ball throw (MBT) measured the explosive force of the extensor muscles of the upper limbs, trunk, and lower limbs. Initial position: The students stood behind a marked line with their feet shoulder-width apart, holding the ball behind their heads with both hands. Procedure: the ball was thrown as far as possible and the participants stepped forward over the line after the ball was released. Assessment: The participants had three attempts; the best score was registered (centimeters).

***50 m race*** (50MR). This measured the displacement speed, maximum cyclic speed, or alactic anaerobic power. Initial position: the students stood behind the starting line of a prepared track. Procedure: At the sound of the word “ready”, the arm of the evaluator was raised and the students adopted an alert position. At the sound of the word “go”, the arm of the evaluator descended, the running began, and a stopwatch was started. Students attempted to run the 50 m as fast as possible without slowing down. Ending: when the finish line was crossed, the timer was stopped. Assessment of the test: the students repeated the test 2 times with a 10 m break between them; the best time of the two attempts made was recorded.

***Coordination test***. Eye–hand coordination test (E-HCT). Participants had to throw a ball to a vertical wall with one hand and catch the ball correctly with the other hand as frequently as possible in 30 s. The participants were free to use overhand, underhand, or a combination of both for the throwing and catching. Subsequently, the participants were able to use their best motor performance strategies. The participants practiced throwing and catching the ball six times before the first attempt.

### 2.4. Data Analysis

All data were analyzed using the statistical package SPSS, version 27.0 (IBM Co., Ltd., Chicago, IL, USA). Descriptive analyses were performed and linear mixed models were used because the standard errors of the estimated model parameters were not underestimated (reducing the risk of inflation of type I errors) and missing data were not a problem [23]. The outcome variables (level 1) were nested for the participants (baseline). Linear mixed model analyses with maximum likelihood (ML) estimates were carried out following the procedures of Snijders and Bosker [24]. A total of eighteen models were fitted for the PA and fitness dimensions, taking the variances for level 1 (repeated measures) and level 2 (participants). The amount of variance explained was calculated using intra-class correlation coefficients (ICCs), which identified the proportion of variance and validated the importance of using multilevel analyses [25].

The models were tested taking VPA, MPA, LPA, and the METs as the dependent variables and linear time (1 = baseline/T1/first year after confinement, 2 = T2/second year after confinement), sex (1 = male, 2 = female), and age as the predictor variables. The models were also tested taking the fitness dimensions as the dependent variables and the following as the predictor variables: Model A—linear time (1 = baseline/T1/first year after confinement, 2 = T2/second year after confinement), sex (1 = male, 2 = female), and age; Model B—linear time (1 = baseline/T1, 2 = T2), sex (1 = male, 2 = female), age, PA (VPA, MPA, LPA, LPA, METs, and regularity), and belonging to a sports club (1 = yes, 2 = no).

## 3. Results

### 3.1. Descriptive Analysis

Table 1 shows the mean scores for men and women in the two time points for the variables analyzed. At both T1 and T2, the females scored higher in the flexibility test; in the rest of the variables, the males scored better, except in VPA (T1), MPA, LPA, and the METs (T1). The highest effect size, both at T1 and T2, was observed in the 3 kg MBT test, followed, at T1, by the results of the CN test and at T2 by the results of the SLJ test.

### 3.2. Multilevel Analysis

Linear mixed analyses (Table 2) showed that when holding sex and age constant, VPA and the METs significantly increased (*p* < 0.001) between T1 and T2 whereas MPA and LPA remained stable. Table 2 provides the covariance parameter estimates; i.e., the parameter estimates associated with the random effects of the model. The within-subject variance was significant in all four-factor models; however, the between-subject variance was not significant in any of them. These results were consistent with the home confinement of the population that was mandatory for all students.

In the physical fitness tests, in Model A (Table 3) (controlled only for the sex and age variables) a growth was observed between T1 and T2 in 5 of the 7 tests: MBT, sit-ups, sit-and-reach, CN, and E-HCT. When the variables of PA and membership in a sports club were included in Model B, the growth between T1 and T2 disappeared in the LBM and NC tests. In these two cases, minutes/week of VPA and PA performed in a sports club largely mitigated the effects of confinement. VPA was significant in three of the models tested: MBT, CN, and E-HCT; and membership in a sports club, in five: MBT, sit-ups, CN, SLT, and E-HCT. Gender was significant in all the models tested: males scored better in all the tests than females, except flexibility. Finally, in the 50 m speed test (Models A and B) and the CN test (Models A), no significant inter-subject variability was observed.

## 4. Discussion

One purpose of this study was to analyze the changes in physical activity between the beginning of the first academic year after a confinement (November 2020) and the beginning of the second academic year post-confinement (“new normality”: November 2021) in a cohort of adolescents living in a rural population in Spain. The results of this study indicated that VPA and the METs significantly increased between the two periods whereas MPA and LPA remained stable. Several studies have detected a decrease in physical activity due to confinements [12,13,14], especially among children who participated in organized team sports [15]. The social restrictions raised concerns about the health of the general population, including cardiovascular and muscle fitness [26,27]. Although a negative trend in the physical activity levels of children was detected after the first year since the beginning of the pandemic [28], this result was encouraging because suggested a positive trend and seemed to suggest that PA levels might return to normal values (i.e., pre-pandemic).

Examining the evolution of physical activity and fitness in adolescents is relevant in this context because both physical activity and fitness are positively related to health [6,29]. Due to the COVID-19 pandemic and the lockdown measures, changes in physical fitness were expected [30]; several studies detected that children exhibited significant physical performance losses during the COVID-19 lockdown [20]. Therefore, another purpose was to analyze the evolution of physical fitness from the beginning of the first year post-confinement to the beginning of the second year post-confinement, after controlling for sex and age.

The results also indicated a growth between the two periods in several components of fitness. For instance, the scores of the following components were significantly higher at the beginning of the second year post-confinement: upper body power, strength endurance (abdominal and hip muscles), cardiovascular fitness, flexibility, and eye–hand coordination. These results suggested that during the confinement, physical fitness and coordination could have been negatively affected due to the quarantine decreed by the government [19,20]. However, when the general population and adolescents were allowed to go outside and practice their sports and physical activities, the growth curve of physical fitness could return to normal values. This would be consistent with other studies [20] and coherent with the notion that physical activity and the practice of sports might lead to an increased physical fitness of the participants [19]. In this regard, López-Bueno et al. [19] observed no significant changes in cardiovascular fitness before and after the quarantine. Specifically, they examined the Vo2 max from November 2019 to November 2020 (192 days after the end of the relaxed confinement). López-Bueno et al. [19] provided explanations for the small differences found. For instance, the researchers indicated that Vo2 max may increase in adolescents due to physical growth and development processed per se; thus, a deceleration of the Vo2 max was plausible. Moreover, there was a relevant period of several months from the end of the confinement and the second Vo2 assessment. As adolescents were allowed to undertake physical activity outdoors again, the detrimental effects of the COVID-19 confinement over the Vo2 max could have been mitigated. Regarding the sex differences, the results of this study were consistent with prior research [31]. At both T1 and T2, males scored higher than females in all tests, except for flexibility (with larger effect sizes in all cases).

The results of the present study provide new information because gender, age, and physical activity performed during the first and the second year post-confinement were controlled in the statistical analysis. Thus, a second model was tested where the variables of physical activity and membership in a sports club were included. The results indicated that the growth between T1 and T2 disappeared in the MBT and CN tests. In these two cases, minutes/week of VPA and PA performed in a sports club largely mitigated the effects of confinement. Therefore, the results of this study were in line with the findings of López-Bueno et al. [19], suggesting that the increase in physical fitness could be due to the physical growth and development processes that happen in normal periods of life. However, regarding cardiovascular and muscle fitness, belonging to a sports club—and, therefore, getting involved in higher levels of VPA—seems to better explain the growth of these components of physical fitness. On the other hand, flexibility, strength endurance of the abdominal muscles, and coordination were not explained by these two variables. It is possible that those students who were involved in more VPA since the beginning of the pandemic experienced increased upper body muscular power and aerobic cardiovascular endurance due to their commitment to a sports club. Prior research observed that adolescents who used to participate in organized team sports reduced their physical activity levels after the lockdown measures [15]. Fortunately, returning to sports activity after the social distancing measures seems to be the best explanatory variable of the growth in cardiovascular and muscle fitness. This result is important because of all the physical fitness components, cardiovascular fitness is the most strongly associated with health outcomes [32] and points out the importance of being involved in a sports club to reach adequate vigorous physical activity levels.

This study provides a new insight into how COVID-19 home confinements and other social distancing measures have affected physical fitness in a sample of Spanish adolescents. After one year of less restrictive social distancing measures, MVPA increased as well as a few capacities of fitness, particularly upper body muscular power, strength endurance, flexibility, cardiovascular fitness, and eye–hand coordination. However, it should be highlighted that after controlling for physical activity and belonging to a sports club, the results indicated that getting involved in more VPA and the commitment to a sports club better explained the development in upper body muscular power and cardiovascular fitness. Long periods of free movement restrictions may negatively affect cardiorespiratory fitness and health [20,30]. However, the maintenance of physical activity due to sports club duties seems to have acted as a protector against the losses or the delay in the normal development of physical fitness during the pandemic. Although it is common for fitness test scores to progress in conjunction with age, the adolescents who were involved in more VPA in this study appeared to be less drastically affected. Thus, the results of this study suggest that membership in a sporting club and a commitment to training—which, subsequently, is associated with higher levels of VPA—might have mitigated the potential negative effect of the social distancing measures.

There are a few limitations of this study that must be addressed. First, this study did not provide a baseline for the impact of COVID-19 on adolescents, but examined the evolution of physical activity and physical fitness between two periods (i.e., after the COVID-19 lockdown and one year afterward). Second, the participants came from a rural environment and a generalizability to other contexts cannot be established. Another limitation of this study was that the physical activity measures were obtained via a questionnaire; data collected through accelerometers could enrich the results. As the pandemic and school closures began in early March 2020, having a pre-test of physical activity closer to this date would have been ideal. However, it is important to note the radical reductions in physical activity reported in other studies due to the strict lockdown measures. Finally, field tests were employed in this study to assess physical fitness; laboratory measures could provide more valid results. Overall, this study provided preliminary data exemplifying the evolution in physical activity and physical fitness of adolescents after the lockdown measures due to the COVID-19 pandemic. Future research should explore the long-term effects of these variables.

## 5. Conclusions

Previous research has observed that muscle and cardiovascular fitness decreased or its evolution was paralyzed in adolescents during the 2019–2020 school years. The results of this study are encouraging and suggest that both males and females tended to recover their physical fitness two years after the lockdown measures. The results indicated a growth between the first and the second year of the pandemic in five of the seven fitness tests. The findings indicated both males and females exhibited mean increases in upper body muscular power, strength endurance of the abdominal muscles, flexibility, cardiovascular fitness, and eye–hand coordination. However, the relevance of physical activity in the physical fitness of adolescents should be highlighted. The results suggested that being involved with sports clubs and performing higher levels of VPA seemed to be related to better cardiovascular and muscle fitness after the two years since the beginning of the so-called “new normality”. As the effects of COVID-19 could be prolonged, future research could focus on understanding why sports participation saw a greater association with these health-related fitness markers. This would be beneficial in this context in order to propose effective solutions to encourage physical activity and membership in sports clubs or associations. Public support for children and families should be considered to encourage the positive effects of physical activity in youths, and guidelines to ensure the maintenance of physical activity in adolescents should be established if similar situations are repeated in the future.

## Figures and Tables

**Table 1:** Descriptive analysis of the study variables and sex differences at T1 and T2.

	T1				ES1	T2				ES2
	Male		Female			Male		Female		
	*M*	*SD*	*M*	*SD*		*M*	*SD*	*M*	*SD*	
VPA	85.76	121.66	83.72	117.42	0.02	192.40 ***	202.25	89.05	117.92	0.72
MPA	134.12	218.87	101.78	145.99	0.17	152.05	186.03	110.21	147.66	0.25
LPA	177.79	342.79	218.21	323.94	−0.12	227.25	395.06	288.26	404.50	−0.15
MET	1807.23	2071.82	1811.80	1902.30	−0.00	2907.72 *	2935.65	2117.59	2258.14	0.30
MBT 3 kg	5.79 ***	1.50	4.22	0.74	1.33	5.93 ***	1.46	4.36	0.70	1.37
Sit-up 30 s	24.52 ***	6.01	20.14	5.49	0.76	30.40 *	11.98	27.28	10.48	0.27
SLJ	1.73 ***	0.30	1.50	0.22	0.87	1.74 ***	0.29	1.45	0.21	1.15
50 m sprint test	8.18 **	1.00	10.01	7.56	−0.36	8.09 ***	0.94	9.42	2.23	−0.78
Sit-and-Reach	22.13	8.86	31.08 ***	8.85	−1.01	23.47	9.26	31.64 ***	9.77	−0.86
Course Navette	5.91 ***	2.22	4.00	1.30	1.05	6.18 ***	2.35	4.27	1.51	0.97
E-HCT	14.85 ***	5.26	11.48	4.21	0.71	18.59 ***	6.34	13.01	4.40	1.02

ES: effect size; VPA: vigorous physical activity; MPA: moderate physical activity; LPA: light physical activity; MET: metabolic equivalent of task; MBT: medicine ball throw test; SLJ: standing long jump test; E-HCT: eye–hand coordination test. * *p* < 0.05, ** *p* < 0.01, *** *p* < 0.001.

**Table 2:** Multilevel analysis of the physical activity levels of the students.

	VPA		MPA		LPA		MET	
	Estimation	*EEM*	Estimation	*EEM*	Estimation	*EEM*	Estimation	*EEM*
*Mixed effects*								
Interception	155.23 ***	22.84	179.53 ***	27.38	222.05 ***	17.61	1804.64 ***	149.66
Time								
T2	62.91 ***	0.06	-		-		745.66 ***	219.97
T1 (baseline)	-		-		-		-	
Age	-		-		-		-	
Sex	−45.79 ***	0.14	−37.39 *	17.73	-		-	-
*Random effects*								
Intra-variance	19,789.53 ***	2063.12	30,013.08 ***	3358.16	132,340.49 ***	13,180.91	4,959,414.03 ***	507,329.87
Between-variance	1130.59	1632.91	2355.49	2819.17	636.65	9638.54	374,921.35	396,585.59
ICC	5629.67		5779.42		6.28		7816.68	

VPA: vigorous physical activity; MPA: moderate physical activity; LPA: light physical activity; MET: metabolic equivalent of task. * *p* < 0.05, *** *p* < 0.001.

**Table 3:** Multilevel analysis of the physical fitness tests of the students.

**(a)**								
	**MBT 3 Kg**		**Sit-Ups 30 s**		**SLJ**		**50 m Sprint Test**	
	**Estimation**	***EEM***	**Estimation**	***EEM***	**Estimation**	***EEM***	**Estimation**	***EEM***
*Mixed effects*								
Interception	7.58 ***	0.23	27.95 ***	1.44	2.02 ***	0.05	6.52 ***	0.34
Time								
T2	0.32 ***	0.06	6.66 ***	0.77	-		-	
T1 (baseline)	-		-		-		-	
Age	-		-		-		-	
Sex	−1.75 ***	0.14	−3.67 ***	0.91	−0.26 ***	0.03	1.58 ***	0.43
*Random effects*								
Intra-variance	0.27 ***	0.03	53.31 ***	6.35	0.02	0.01	17.41 ***	1.66
Between-variance	1.13 ***	0.12	17.95 **	6.27	0.05	0.01	0.37	1.18
ICC	1076.74		2846.72		6.28		2268.78	
*Mixed effects*								
Interception	8.00 ***	0.27	31.46 ***	1.73	2.14 ***	0.05	7.98 ***	
Time								
T2	-		6.19 ***	0.78	-		-	
T1 (baseline)	-		-		-		-	
Age	-	0.01	-		-		-	0.02
Sex	−1.57 ***	0.15	−2.79 **	0.92	−0.22 ***	0.03	1.53 ***	0.51
PA								
VPA	10 *	0.05	-		-			
MPA	-		-		-			
LPA	-		-		-			
MET	-		-		-			
Regularity	-		-		-		−1.39 **	0.51
Sports club	−0.35 **	0.12	−3.15	0.90	−0.11 ***	0.02		
*Random effects*								
Intra-variance	0.29 ***	0.02	54.58 ***	6.46 ***	0.02 ***	0.00	16.71 ***	0.18
Between-variance	0.91 ***	0.06	14.09 *	6.08	0.04 ***	0.01	0.76	0.12
ICC	873.55		2841.99		−6.99		2268.52	
**(b)**								
	Sit-and-Reach		Course Navette			Eye–Hand Coordination		
	Estimation	*EEM*	Estimation		*EEM*	Estimation		*EEM*
*Mixed effects*								
Interception	13.41 ***	1.69	7.87 ***		0.35	19.56 ***		0.94
Time								
T2	1.43 **	0.49	0.35 ***		0.09	3.37 ***		0.34
T1 (baseline)	-		-			-		
Age	-		-			-		
Sex	8.76 ***	1.09	−1.85 ***		0.23	−4.39 ***		0.60
*Random effects*								
Intra-variance	17.82 ***	2.19	0.60 ***		0.08	9.47 ***		1.15
Between-variance	64.95 ***	7.01	3.02		0.31	17.01 ***		2.23
ICC	2793.90		1479.18			2409.07		
*Mixed effects*								
Interception	13.41 ***	1.69	8.85 ***		0.37	20.28 ***		0.26
Time								
T2	1.43 **	0.49	-			2.97 ***		0.36
T1 (baseline)	-		-			-		
Age	-		-			-		
Sex	8.76 ***	1.09	−1.53 ***		0.22	−3.88 ***		0.62
PA								
VPA	-		0.15 *		0.06	0.50 *		0.21
MPA	-					-		
LPA	-					-		
MET	-					-		
Regularity	-					-		
Sports club	-		−0.91 ***		0.16	−1.12 *		0.53
*Random effects*								
Intra-variance	17.82 ***	2.19	0.66 ***		0.09	9.34 ***		1.15
Between-variance	64.95 ***	7.01	2.54 ***		0.28	16.34 ***		2.18
ICC	2793.90		1461.78			2405.53		

ES: effect size; VPA: vigorous physical activity; MPA: moderate physical activity; LPA: light physical activity; MET: metabolic equivalent of task; MBT: medicine ball throw test; SLJ: standing long jump test. * *p* < 0.05, ** *p* < 0.01, *** *p* < 0.001.

## Data Availability

Data are available for research. Any further inquiries can be directed to the authors.
